# Case Report: Replacement of PD-1 inhibitors with PD-L1 inhibitors in the treatment of squamous non-small-cell lung carcinoma

**DOI:** 10.3389/fimmu.2023.1243980

**Published:** 2023-08-15

**Authors:** Tong Wu, Yujun Li, Xiaonan Cui, Chunxia Zhang

**Affiliations:** Department of Oncology, The First Affiliated Hospital of Dalian Medical University, Dalian, China

**Keywords:** tislelizumab, cardiotoxicity, sugemalimab, PD-L1 inhibitor, squamous NSCLC, case report

## Abstract

**Background:**

Immune checkpoint inhibitor (ICI)-associated cardiotoxicity is a relatively uncommon immune-related adverse effects (irAEs) with a high mortality rate. There are few recommendations for the replacement of different immune checkpoint inhibitors in domestic and international reports.

**Case presentation:**

We report a case of a patient with squamous non-small cell lung carcinoma (squamous NSCLC) who developed cardiotoxicity after being treated with a programmed death-1 (PD-1) inhibitor and then changed to a PD-L1 inhibitor to continue the treatment. A significant benefit was observed after four cycles of immunotherapy, and no further cardiotoxicity occurred after the treatment was started.

**Conclusion:**

This case demonstrates that myocardial damage induced by tislelizumab (PD-1 inhibitor) can be improved after switching to sugemalimab (PD-L1 inhibitor) and that antitumor immunotherapy is effective. This result may have important implications for optimizing immunotherapy management regimens in cancer patients.

## 1 Introduction

Tislelizumab is a PD-1 inhibitor that enhances the human tumor immune response by specifically blocking the interaction between PD-1 and PD-L1. Based on clinical research evidence from RATIONALE 307, tislelizumab was approved by the National Medical Products Administration (NMPA) for the first-line treatment of advanced squamous NSCLC on 12/01/2021 (1). However, there were no clinical data and treatment included in the trial related to tislelizumab combination chemotherapy-related cardiotoxicity. This article reported a case of a squamous NSCLC patient who developed cardiotoxicity after receiving one cycle of combination therapy using tislelizumab (PD-1 inhibitor), combined with nab-paclitaxel and carboplatin. Moreover, the combination therapy continued after the patient got better while tislelizumab was replaced with sugemalimab (PD-L1 inhibitors).

## 2 Case description

In March 2022, a 59-year-old man was admitted to the clinic with a persistent cough producing blood-streaked sputum for 6 months, which worsened over the last 2 months. He arrived with stable vital signs, without chest heartburn or pain, and without dizziness or palpitations (ECOG score: 1). The patient had a history of coronary heart disease for 10 years with one stent implanted in the circumflex, and cardiac ultrasound indicated that cardiac function was lower than normal. There was no previous history of hypertension or diabetes mellitus. CT scan with chest contrast revealed a right hilar mass enveloping the right pulmonary artery, and the right hilar lymph node was about 2.3 cm. A pathological tissue biopsy was performed by tracheoscopy to consider squamous carcinoma, which was finally diagnosed as stage IIIB squamous carcinoma of the right lung (pT4N2M0), and no surgical indication was available at this stage. A craniocerebral MRI enhancement scan and abdominal CT non-contrast scan did not reveal metastases. Cardiac ultrasound showed that the left ventricular ejection fraction (LVEF) was reduced with 50%. In addition, the electrocardiogram (ECG), cardiac enzymes, and liver and kidney functions were normal.

**Figure 1:**
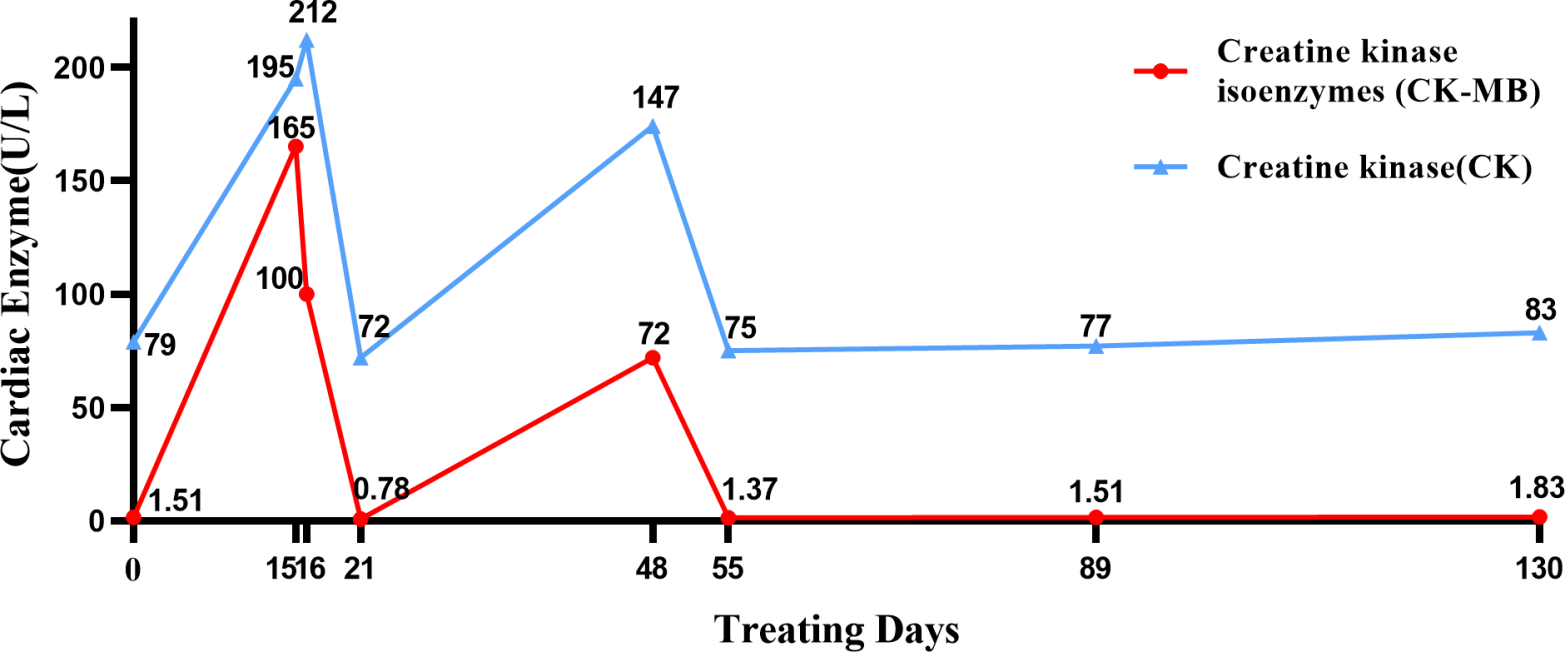
Timeline of cardiac enzyme changes. In this figure, we charted the changes in cardiac enzyme levels during the treatment from the day of the patient’s first chemotherapy as the starting point for calculation.

Subsequently, the patient was treated with nab-paclitaxel 300 mg + carboplatin 400 mg + tislelizumab (PD-1 inhibitor) 200 mg for one cycle. The treatment went well, and the patient had no discomfort. Two weeks later, the patient’s cardiac enzymes were significantly elevated on clinic recheck: creatine kinase (CK) 195 U/L (reference: 0–173), CK isoenzyme 165 U/L (reference: 0–24), lactic dehydrogenase (LDH) 238 U/L (reference: 15–220). We considered the development of immunotherapy-related cardiotoxicity (G2); thus, the patient was administered trimetazidine to support myocardial nutrition. On the next day, the cardiac enzymes showed that CK isoenzyme decreased to 100 U/L, CK 212 U/L, and LDH 245 U/L. Cardiac ultrasound showed LVEF 43%, 7% lower than the baseline, so myocardial nutrition therapy was continued. Before the second cycle of treatment, all the cardiac enzymes returned to normal on recheck (**Figure 1**).

**Figure 2:**
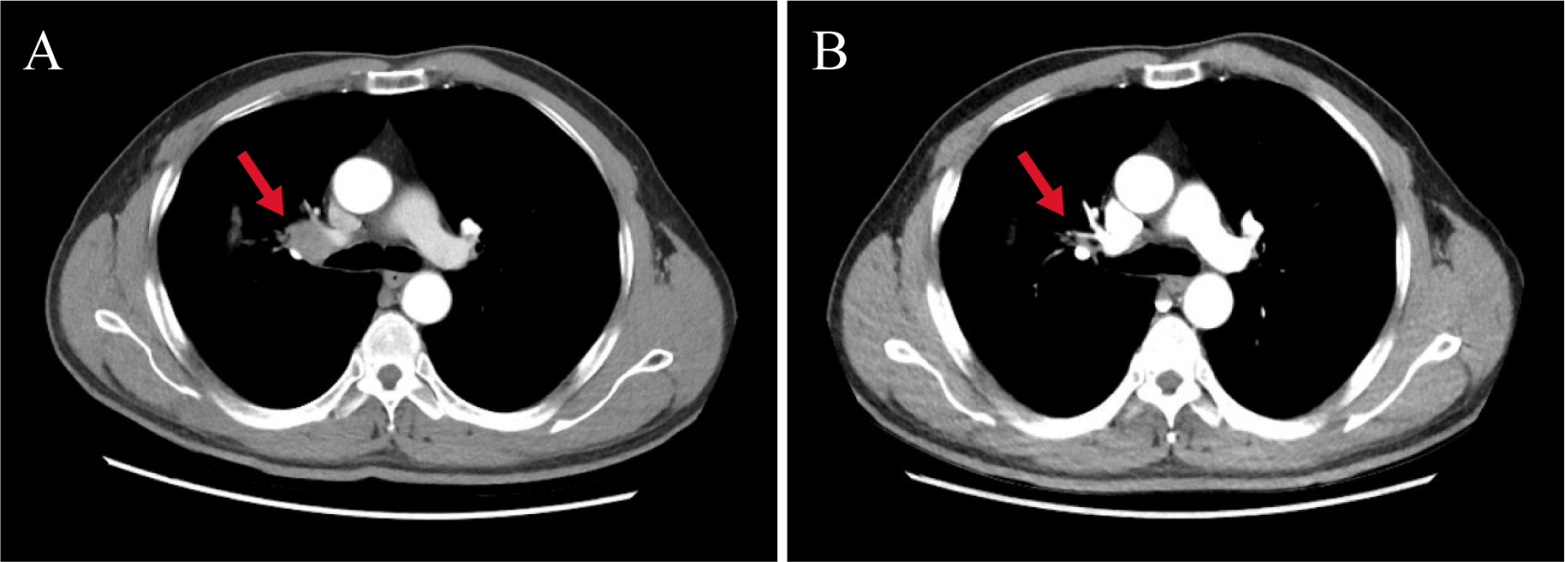
Changes in target lesion size during treatment. **(A)** CT scan with chest contrast (before immunotherapy). **(B)** CT scan with chest contrast (after 4 months of sugemalimab treatment). The red arrows in the figure mark the target lesions of enlarged lymph nodes. Chest imaging showed a reduction in the size of the hilar lymph nodes from 2.3 cm to 1.4 cm after four cycles of treatment, with an assessment of PR at the end of treatment.

After extensive consideration of the necessity of treatment and the differences in the incidence of cardiotoxicity among different types of ICIs (2, 3), we decided to initiate one cycle of chemotherapy combined with PD-L1 inhibitor treatment after the cardiac enzymes had returned to normal. Specific dosage included nab-paclitaxel 300 mg + carboplatin 400 mg + sugemalimab (PD-L1 inhibitor) 1,200 mg. The patient was rechecked after a month; the results of troponin, CK, and LDH were normal, while that of CK isoenzyme was mildly increased (72 U/L). Four cycles of sugemalimab combined with chemotherapy later, cardiac enzymes and ECG remained normal, and LVEF increased from 43% to 55%. Treatment is currently ongoing. Tumor markers including carcinoembryonic antigen (CEA, from 5.45 ng/mL to 3.78 ng/mL), cytokeratin 19 fragment (CYFRA21-1, from 7.3 ng/mL to 2.21 ng/mL), and squamous cell carcinoma-associated antigen (SCC, from 1.05 ng/mL to 0.65 ng/mL) gradually decreased during the treatment. Review of CT scan with contrast for evaluation of the target lesion prompted the efficacy of the final treatment as PR (**Figure 2**). Subsequently, the patient continued to be followed up and the lesion remained stable without recurrence of cardiotoxicity.

## 3 Discussion

In this case, the patient had a history of coronary artery disease, which increased the risk of immunotherapy-related cardiotoxicity (4). The patient developed cardiotoxicity after one cycle of tislelizumab combined with chemotherapy, and the subsequent treatment options were adjusted from tislelizumab to sugemalimab. During this period, the patient’s cardiotoxicity returned to normal while the tumor lesions were well controlled. It suggests that the adverse reaction spectrum may be different between PD-1 inhibitors and PD-L1 inhibitors. A meta-analysis comparing irAEs between PD-1 and PD-L1 inhibitors in multicancer clinical studies included 125 clinical trials with a total of 20,128 patients. The analysis showed that the overall incidence of adverse reactions was lower in PD-L1 inhibitors than in PD-1 inhibitors and the incidence of adverse reactions (>grade 3) was significantly lower in PD-L1 inhibitors than in PD-1 inhibitors (RR: 1.58; 95% CI: 1.00–2.54) (3).

We have considered that it may depend on the different mechanisms of PD-1 inhibitors compared with PD-L1 inhibitors. It has been reported that PD-1 inhibitors simultaneously block the binding of PD-1 on the T-cell surface to PD-L1/2 on the immune cell surface, which increases the risk of potential autoimmune reactions (5). In contrast, PD-L1 inhibitors are able to preserve the immunomodulatory function of the PD-1/PD-L2 pathway and reduce the risk of irAEs. Indeed, the mechanism of ICI-associated cardiotoxicity is not yet clear, and it has been suggested to be possibly associated with infiltration of T cells and macrophages (6). After comparing different PD-L1 inhibitors, we proposed the possible hypothesis that sugemalimab was able to block the binding of PD-L1 to PD-1, resulting in an increased binding of PD-L2 to PD-1, which preserved the immunosuppressive effect of PD-L2. Meanwhile, it also blocks the binding of PD-L1 to CD80, which liberates CD80 and increases the binding of CD80/CTLA-4, exerting immunosuppressive effects and attenuating immune-related toxic reactions. As a unique property of sugemalimab, it activates antibody-dependent cell-mediated phagocytosis (ADCP) *via* binding the Fc segment of the antibody to receptors on the surface of macrophages, inducing further destruction of tumors and resulting a better immunotherapeutic effect. In addition, sugemalimab is a full-length, fully human PD-L1 targeted immunoglobin with lower immunogenicity, which is one of the reasons why it has a lower incidence of irAE (7). Tislelizumab exerts a blocking effect on PD-1 and inhibits both PD-L1 and PD-L2 pathways, causing PD-L1 overexpression. That depletes CD80, making the immunosuppressive effect of the related pathway diminished. At the same time, increased binding of PD-L2 to repulsive guidance molecule B (RGMB) stimulates T-cell activation and may induce autoimmune responses (5, 8). Previous studies have indicated that cardiomyocytes develop immune tolerance mainly by upregulating the expression of PD-L1 to protect cardiomyocytes from immune system attack (9). In a follow-up study of ICI-treated patients, the incidence rates of myocarditis related to PD-1 inhibitors and PD-L1 inhibitors were 0.5% and 2.4%, respectively. Hence, the damage caused by PD-L1 inhibitors to cardiomyocytes may be significant. However, the patient in this case showed less cardiotoxicity after receiving sugemalimab than before and did not develop severe myocardial damage. This result was different from the previous data but was consistent with results obtained from some clinical trials. In a large meta-analysis including 19,217 patients, the incidence of cardiac adverse events was shown to be 33% in anti-PD-1 (n = 9136, 4/12) and 12% in anti-PD-L1 (n = 3164, 3/25) (10). Although certain clinical trials have shown that PD-L1 inhibitors have a lower overall incidence of adverse events than PD-1 inhibitors (3), there are limited studies on mitigating irAE by transitioning PD-1 inhibitors to its ligands. Thus, we can only speculate on the benefits of such therapy from the clinical data. In fact, this case is based on the individual specificity of the patient, and whether the conclusion is of broad significance needs to be pondered. Further research data is necessary to substantiate this point of view. Being a newly approved PD-L1 inhibitor, sugemalimab presents an opportunity for further investigation into whether it possesses its distinct cardioprotective mechanism. However, we need to emphasize that it has no effect on the antitumor effect of the drug after changing the type of ICIs. Immunotherapy is a double-edged sword, and it will be the major challenge to figure out how to use the variations in mechanisms between different types of ICIs to enhance immunotherapy treatment regimens for cancer patients.

In summary, the incidence of cardiotoxicity is <1% in the irAE spectrum (11), including cardiomyopathy (mainly myocarditis), pericardial effusion, arrhythmias, acute coronary syndrome, and heart failure (12). According to previous clinical trials and retrospective studies, the mortality rate of myocarditis was as high as 39.7%–50% (10, 13). The management of ICI-related cardiotoxicity in the latest domestic and international guidelines recommends corticosteroids as the first-line immunosuppressive drug for ICI-related myocarditis (9), and it is based on AE grading and AE recovery to decide whether to restart the original regimen of immunotherapy. Unfortunately, the lack of recommendations for replacing a different type of ICIs has deprived a number of opportunities for immunotherapy. We anticipate that the paradigm of continuing treatment with PD-L1 inhibitors rather than PD-1 inhibitors after cardiotoxicity develops will provide the patients additional alternatives.

## Data availability statement

The original contributions presented in the study are included in the article/supplementary material. Further inquiries can be directed to the corresponding authors.

## Ethics statement

Written informed consent was obtained from the individual(s) for the publication of any potentially identifiable images or data included in this article. Written informed consent was obtained from the participant/patient(s) for thepublication of this case report.

## Author contributions

TW and YL collected of data and drafted of the manuscript. CZ and XC revised the manuscript critically for important intellectual content and gave final approval of the manuscript submitted.
